# Supplementary material for: The deubiquitinase USP17LA negatively regulates T-cell activation and attenuates anti-tumor immunity
Source: EMBO Rep. 2025 Sep 29;26(21):5300–25. doi: 10.1038/s44319-025-00584-5 (PMC12592519; doi:10.1038/s44319-025-00584-5)
Supplement: Supplementary file 10 — Expanded View Figures [file 44319_2025_584_MOESM10_ESM.pdf]

Expanded View Figures

Figure EV1. Transcriptomic analysis of activated EL4 cells, sequence homology alignment of the USP17L family, and cDNA-based genotyping of *Usp17la* and *Usp17lb* knockout mice.

(**A**) GO pathway analysis of upregulated expressed genes in PMA plus ionomycin vs untreated EL4 cells by RNA-seq were conducted using the David database (https://david.ncifcrf.gov/). (**B**) KEGG pathway analysis of upregulated expressed genes in PMA plus ionomycin vs untreated EL4 cells by RNA-seq were conducted using the David database (https://david.ncifcrf.gov/). (**C**) Alignment was performed using the Clustal Omega program and reformatted with the MView tool. cov, percent coverage; pid, percent identity with respect to the first sequence (USP17LA). Identities normalized by aligned length. Colors indicate identical sequence. (**D**, **E**) Schematic diagram of cDNA-based genotyping for *Usp17la* and *Usp17lb* knockout mice. $CD3^+$ T cells were sorted from mouse spleens, followed by RNA extraction, reverse transcription, and genotyping using cDNA. (**F**, **G**) Representative RT-PCR results showing cDNA-based genotyping of $Usp17la^{+/+}$ vs. $Usp17la^{-/-}$ and $Usp17llb^{+/+}$ vs. $Usp17lb^{-/-}$ transcripts using RNA from $CD3^+$ T cells sorted from mouse spleens. (**H**) Genotypic distribution of 522 offspring from $Usp17la^{+/-}$ × $Usp17la^{+/-}$ crosses was compared with the expected Mendelian ratio (1:2:1) using a χ^2 goodness-of-fit test. No significant deviation was observed ($P > 0.05$). (**I**) Body weight of 8-week-old $Usp17la^{+/+}$, $Usp17la^{+/-}$, and $Usp17la^{-/-}$ mice ($n = 5$ per genotype). (**J**) Gross appearance of 8-week-old $Usp17la^{+/+}$ and $Usp17la^{-/-}$ mice. Data are representative of two independent experiments (**I**). Data are shown as mean ± s.d. Statistical analyses were performed using one-way ANOVA with Sidak's multiple comparisons test (**I**). NS, not significant.

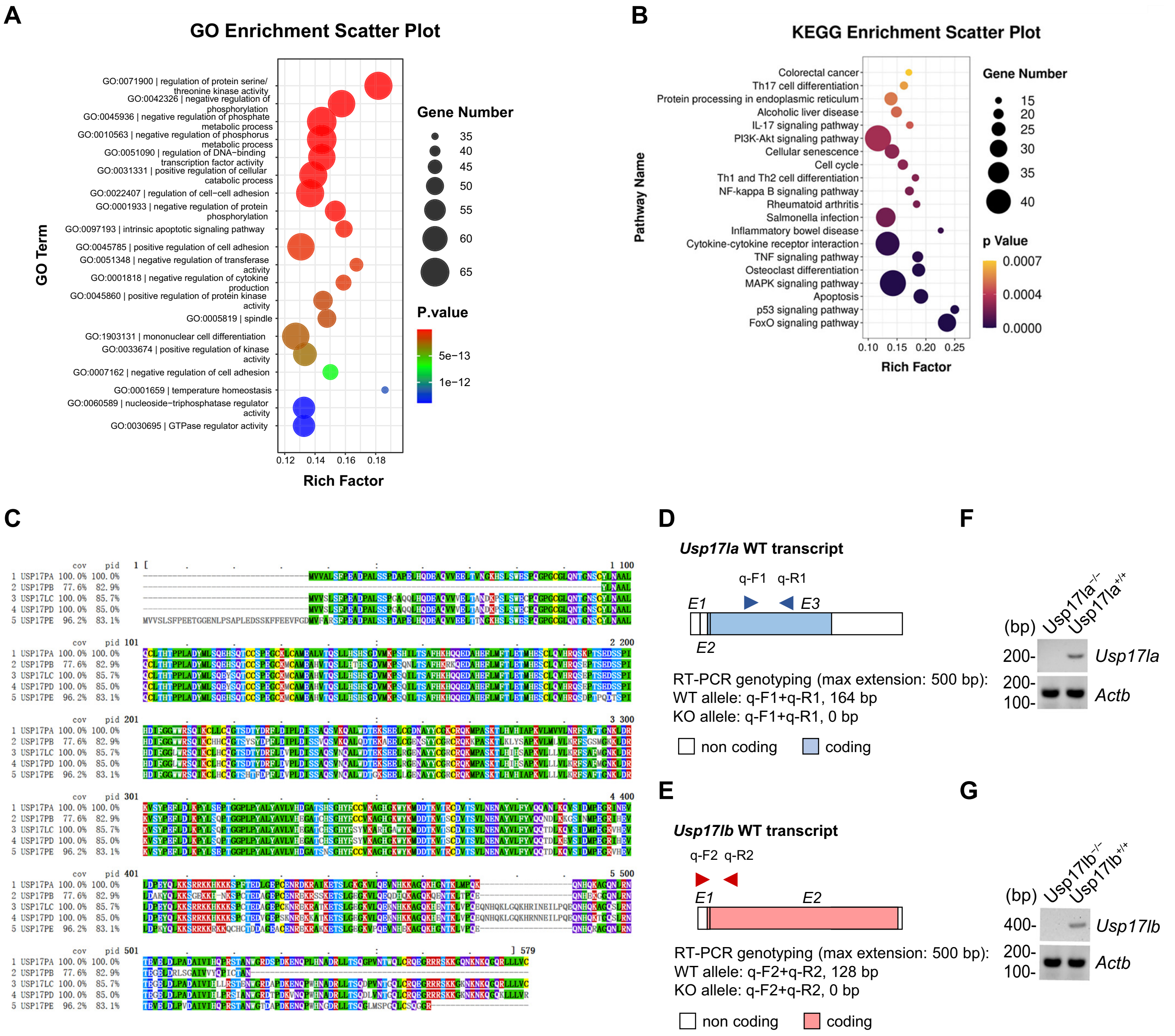

H

♂ *Usp17la*+/− x ♀ *Usp17la*+/−

Genotype	Observed♦	Expected
Usp17la+/+	133	130.5
Usp17la+/−	251	261
Usp17la−/−	138	130.5

♦Observed: P>0.05

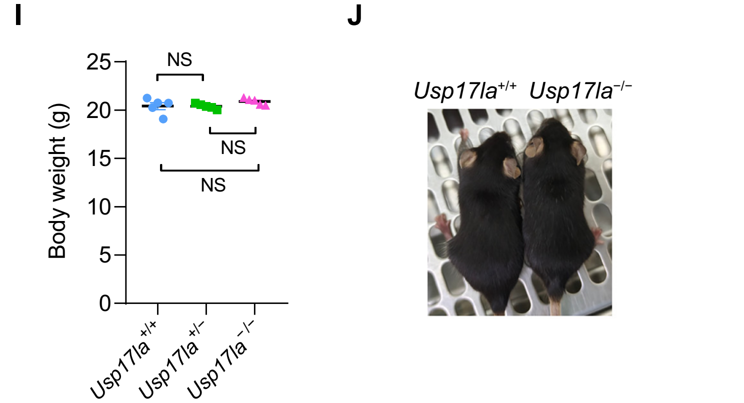

 © The Author(s)

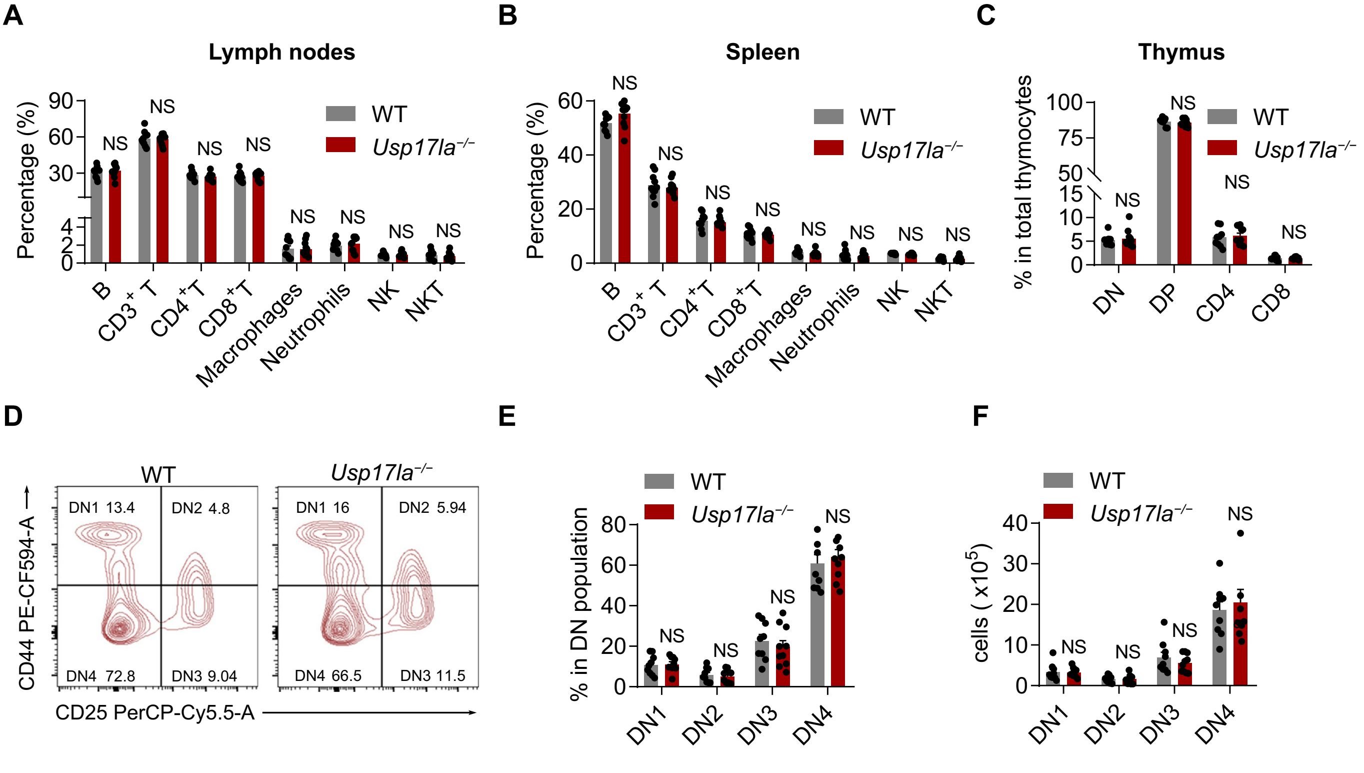

Figure EV2. The percentage of immune cells in lymphoid organs and DN subpopulation in *Usp17la*$^{-/-}$ mice.

(**A**, **B**) The percentage of B, CD3^{+} T, CD4^{+} T, CD8^{+} T, macrophages, neutrophils, NK and NKT cells in lymph nodes and spleen of WT and *Usp17la*$^{-/-}$ mice ($n = 10$–11 per genotype). (**C**) The percentage of DN, DP, CD4 SP, and CD8 SP subpopulations in thymus of WT and *Usp17la*$^{-/-}$ mice ($n = 10$–11 per genotype). (**D**–**F**) Flow cytometry analysis the frequency and number of DN1, DN2, DN3, and DN4 cells in DN population of WT and *Usp17la*$^{-/-}$ mice ($n = 10$–11 per genotype). Data are combination of two independent experiments. Data are shown as mean ± s.d. Statistical analyses were performed using two-way ANOVA followed by Sidak's multiple comparisons test (**A**–**C**, **E**, **F**). NS, not significant.

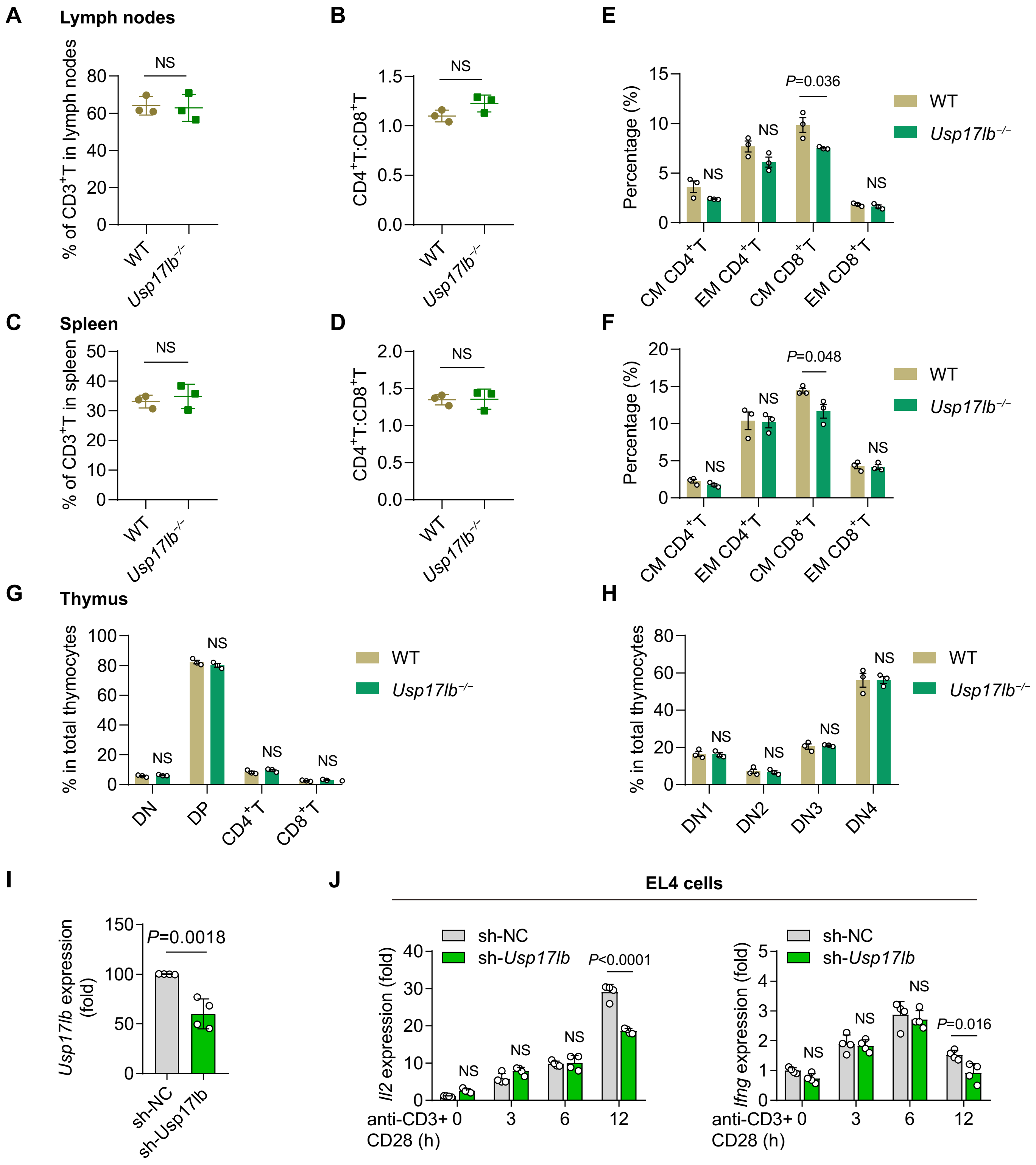

A
Lymph nodes
% of CD3+T in lymph nodes
NS
WT
Usp17lb−/−
B
CD4+T:CD8+T
NS
E
Percentage (%)
P=0.036
NS
CM CD4+T
EM CD4+T
CM CD8+T
EM CD8+T
C
Spleen
% of CD3+T in spleen
D
F
P=0.048
G
Thymus
% in total thymocytes
DN
DP
CD4+T
CD8+T
H
DN1
DN2
DN3
DN4
I
Usp17lb expression (fold)
P=0.0018
sh-NC
sh-Usp17lb
J
EL4 cells
Il2 expression (fold)
P<0.0001
anti-CD3+ CD28 (h)
Ifng expression (fold)
P=0.016

 © The Author(s)

Figure EV3. Immune profiling of *Usp17lb*$^{-/-}$ mice and functional activation of EL4 cells following *Usp17lb* loss.

(**A**, **B**) Percentages of $CD3^+$ T cells in total lymphocytes and the ratio of $CD4^+$ T cells to $CD8^+$ T cells in lymph nodes of WT and *Usp17lb*$^{-/-}$ mice ($n = 3$ per genotype). (**C**, **D**) Percentages of $CD3^+$ T cells in total lymphocytes and the ratio of $CD4^+$ T cells to $CD8^+$ T cells in spleen of WT and *Usp17lb*$^{-/-}$ mice ($n = 3$ per genotype). (**E**, **F**) Frequencies of CM and EM subpopulations in $CD4^+$ T cells or $CD8^+$ T cells in lymph nodes and spleen of WT and *Usp17lb*$^{-/-}$ mice ($n = 3$ per genotype). (**G**, **H**) Flow cytometry analysis the percentages of $CD4^-CD8^-$ (DN), $CD4^+CD8^+$ (DP), $CD4^+CD8^-$ (SP4), and $CD4^-CD8^+$ (SP8) subpopulations in thymus, and $CD44^+CD25^-$ (DN1), $CD44^+CD25^+$ (DN2), $CD44^-CD25^+$ (DN3), and $CD44^-CD25^-$ (DN4) subpopulations among DN thymocytes of WT and *Usp17lb*$^{-/-}$ mice ($n = 3$ per genotype). (**I**) RT-PCR analysis of *Usp17lb* knockdown efficiency in EL4 cells following infection with sh-NC or sh-*Usp17lb* lentivirus ($n = 4$ per group). (**J**) RT-PCR analysis of the expression of *Il-2* and *Ifng* in EL4 cells infected with sh-NC or sh-*Usp17lb* lentivirus following anti-CD3 plus CD28 stimulation for the indicated times ($n = 4$ per group). Data are representative of three (**A–H**) or two independent experiments (**I, J**). Data are shown as mean ± s.d. Statistical analyses were performed using unpaired, two-tailed Student's *t*-test (**A–D**, **I**) and two-way ANOVA followed by Sidak's multiple comparisons test (**E–H**, **J**). NS not significant.

© The Author(s)

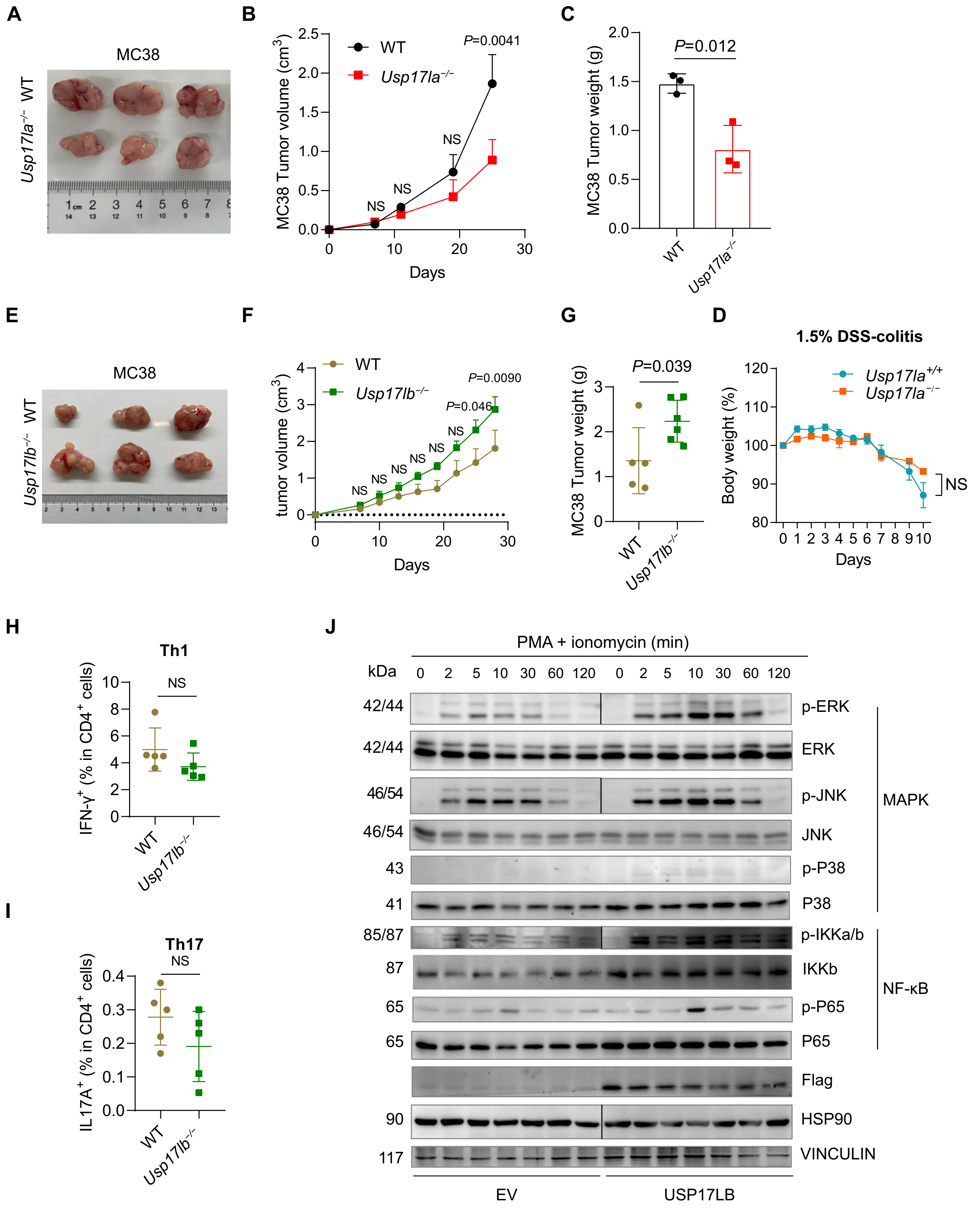
A
MC38
Usp17la−/− WT
B
MC38 Tumor volume (cm3)
WT
Usp17la−/−
P=0.0041
NS
Days
C
MC38 Tumor weight (g)
P=0.012
WT
Usp17la−/−
E
MC38
Usp17lb−/− WT
F
tumor volume (cm3)
WT
Usp17lb−/−
P=0.0090
P=0.046
NS
Days
G
MC38 Tumor weight (g)
P=0.039
WT
Usp17lb−/−
D
1.5% DSS-colitis
Usp17la+/+
Usp17la−/−
Body weight (%)
NS
Days
H
Th1
NS
IFN-γ+ (% in CD4+ cells)
WT
Usp17lb−/−
I
Th17
NS
IL17A+ (% in CD4+ cells)
WT
Usp17lb−/−
J
PMA + ionomycin (min)
kDa
0 2 5 10 30 60 120 0 2 5 10 30 60 120
42/44 p-ERK
42/44 ERK
46/54 p-JNK
46/54 JNK
43 p-P38
41 P38
MAPK
85/87 p-IKKa/b
87 IKKb
65 p-P65
65 P65
NF-κB
Flag
90 HSP90
117 VINCULIN
EV
USP17LB

Figure EV4. *Usp17la* deficiency suppresses tumor growth, whereas *Usp17lb* deficiency promotes it and its overexpression augments TCR signaling.

(**A–C**) Tumor growth was assessed in terms of the tumor volume (**A**), tumor size (**B**), and tumor weight (**C**) in WT and *Usp17la*$^{-/-}$ mice after MC38 colon cancer cells inoculation ($n = 3$ per genotype). (**D**) Body weight changes in DSS-induced colitis. Mice were administered 1.5% DSS in drinking water for 7 days, followed by a 3-day recovery period with DSS-free water. Body weight was recorded over a total of 10 days. ($n = 4$ per genotype). (**E–G**) Tumor growth was assessed in terms of the tumor volume (**E**), tumor size (**F**), and tumor weight (**G**) in WT and *Usp17lb*$^{-/-}$ mice after MC38 colon cancer cells inoculation (for **E**, $n = 3$ per genotype; for (**F**, **G**), $n = 5$–6 per genotype). (**H**, **I**) Flow cytometry analysis of the expression of IFN-γ and IL-17A in CD4^{+} T cells from spleen of WT and *Usp17lb*$^{-/-}$ mice ($n = 5$ per genotype).
(**J**) Immunoblot analysis of TCR signaling-associated proteins in PMA plus ionomycin-stimulated EL4 cells overexpressed with empty control (EV) or Flag-USP17LB. Data are representative of two independent experiments (**A–E**, **H–J**) or pooled from two independent experiments (**F**, **G**). Data are shown as mean ± s.d. Statistical analyses were performed using two-way ANOVA followed by Sidak's multiple comparisons test (**B**, **D**, **F**) and unpaired, two-tailed Student's *t* test (**C**, **G**, **H**, **I**). NS, not significant. Source data are available online for this figure

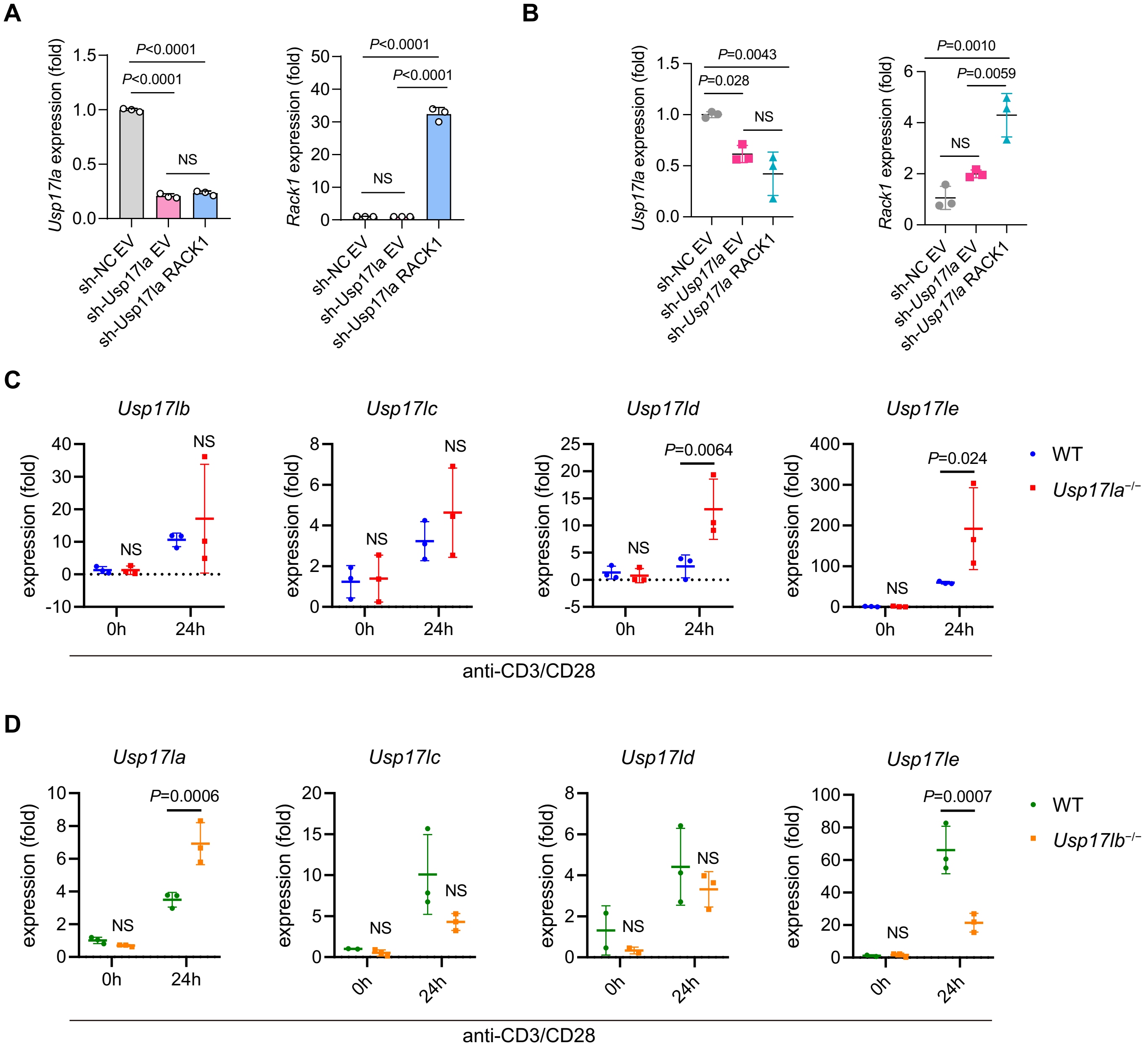

Figure EV5. Compensatory effects induced by *Usp17la* and *Usp17lb* deficiency.

(**A**) RT-PCR analysis of *Usp17la* and *Rack1* mRNA expression in EL4 cells following infection with sh-NC or sh-*Usp17la* in combination with OE-EV or OE-RACK1 lentiviruses ($n = 3$ per group). (**B**) RT-PCR analysis of *Usp17la* and *Rack1* mRNA expression in stably NFAT-luciferase-transduced EL4 cells following infection with sh-NC or sh-*Usp17la* in combination with OE-EV or OE-RACK1 lentiviruses ($n = 3$ per group). (**C, D**) RT-PCR analysis of other *Usp17l* family members in *Usp17la* or *Usp17lb*-deficient cells with or without anti-CD3 plus anti-CD28 stimulation for 24 h ($n = 3$ per group). Data are representative of two independent experiments (**A–D**). Data are shown as mean ± s.d. Statistical analyses were performed using one-way ANOVA with Dunnett's multiple comparisons test (**A, B**) and unpaired, two-tailed Student's *t* test (**C, D**). NS not significant.

 © The Author(s)
